# The era of technology in healthcare: an evaluation of telerehabilitation on patient outcomes—a systematic review and meta-analysis protocol

**DOI:** 10.1186/s13643-023-02248-8

**Published:** 2023-05-04

**Authors:** Sharan Jaswal, Joyce Lo, Gobika Sithamparanathan, Behdin Nowrouzi-Kia

**Affiliations:** 1 Department of Occupational Science and Occupational Therapy, Temerty Faculty of Medicine, University of Toronto, Toronto, ON Canada; 2 Centre for Research in Occupational Safety and Health, School of Rural and Northern Health, Laurentian University, Sudbury, ON Canada; 3 Faculty of Medicine, Rehabilitation Sciences Institute, University of Toronto, Toronto, ON Canada

**Keywords:** Telerehabilitation, Telehealth, Remote healthcare, Occupational therapy, Speech therapy, Physical therapy

## Abstract

**Background:**

The World Health Organization announced the outbreak of the Coronavirus disease as a global pandemic on March 11, 2020. Since then, rapid implementation of telehealth approaches into the healthcare system have been evident. The pandemic has drastically impacted the lives of many around the globe and has detrimentally affected our healthcare systems, specifically with the delivery of healthcare. This has had many implications on rehabilitation services such as, occupational therapy, physiotherapy, and speech therapy. The delivery of mental health services remotely may be referred to as *teletherapy, telemental health, telepsychiatry,* and *telepsychology.* Telerehabilitation has become a necessity over the course of the pandemic due to safety concerns with COVID-19 transmission. The primary aim of this systematic review protocol is to evaluate the literature on the effect of telerehabilitation on patient outcomes and propose directives for future research based on the evidence reviewed.

**Methods:**

A systematic review and meta-analysis will be conducted to examine the literature on the effect of telerehabilitation on patient outcomes following the Preferred Reporting Items of Systematic Reviews and Meta-Analyses (PRISMA) guidelines (PRISMA, 2015). The systematic review will use the following databases to examine the literature on telerehabilitation and patient outcomes: APA PsychINFO, Embase (Ovid), MEDLINE (Ovid), CINAHL, and Scopus.

**Discussion:**

The utilization of telerehabilitation and similar telehealth treatments has increased throughout the COVID-19 pandemic. However, much is still unclear regarding the effectiveness of these methods in the delivery and service of healthcare, and their effect on health outcomes. This review will identify and address the knowledge gaps in the literature, which will provide further directions for future research.

**Trial registration:**

This systematic review has been registered with PROSPERO under registration number CRD42022297849.

**Supplementary Information:**

The online version contains supplementary material available at 10.1186/s13643-023-02248-8.

## Background

There has been a recent acceleration in the use of the term “telehealth” within the past few years, despite its existence since the 1940s [1]. Telehealth is the use of electronic communications to exchange medical information to support patient’s health remotely [2]. The outbreak of the Coronavirus disease (COVID-19) was marked in 2019, and due to the rapid spread and the severity of the disease, the World Health Organization (WHO) declared COVID-19 as a global pandemic on March 11, 2020 [3, 4]. COVID-19 has become a global threat with over six million deaths globally reported to date (April 10, 2023), detriments to economic and social growth, and negative impacts on many lives worldwide [5]. The pandemic has impacted the healthcare system drastically as in-person healthcare delivery has declined due to restrictions to ensure the safety of healthcare workers and patients. However, this has had implications on rehabilitation services, including limiting access to health services and impacting quality of care with those requiring, occupational therapy, physiotherapy, and speech therapy [6]

Telehealth can be considered as a broad umbrella that includes classifications of clinical and non-clinical services delivered remotely [2]. Telerehabilitation is a clinical rehabilitation service that focuses on evaluating, diagnosing, and treating [7]. Like other telehealth classifications, telerehabilitation can be provided and delivered through remote and online services. Remote services arose in response to the pandemic, as they traditionally would be conducted in-person, whereas online services were designed with the purpose to be completed online. These can include asynchronous online visits, virtual check-ins, and telephone evaluations delivered by occupational therapists (OTs), physical therapists (PTs), and speech-language pathologists (SLPs) [7].

The delivery of mental health services remotely may be referred to as *teletherapy, telemental health, telepsychiatry,* and *telepsychology.* A study conducted by the American Psychological Association found that 76% of respondents (clinicians) indicated that they are only providing remote services to their mental health patients [8]. The same percentage of respondents also expressed feeling educated and confident in delivering telehealth services, despite the challenges. An Australian study evaluating the perceived impact of telehealth delivery on the quality of mental health among youth found that most youth stated telehealth interventions had a positive impact on service quality [9]. The study also found that 65% of clinicians demonstrated high levels of interest in providing treatment and care through telehealth services and applications [9]. Matsumoto et al. (2018) conducted a study evaluating the feasibility of videoconference-delivered cognitive behavioural therapy (CBT) compared to face-to-face delivered CBT among adult patients diagnosed with obsessive–compulsive disorder (OCD), panic disorder (PD), or social anxiety disorder (SAD) [10]. The study found a significant decrease in the patient’s mild to severe symptoms for each disorder. There was a 40% reduction in OCD symptoms, 50% for PD symptoms, and 22% for SAD symptoms. The videoconference-delivered CBT yielded positive reactions, with 86% of patients being satisfied with this method, and 83% of patients preferring the videoconference-delivered CBT instead of face-to-face CBT [10].

The majority of PTs adopt a hands-on approach to relieve acute and chronic musculoskeletal pain and improve function and movement of the body. A study in 2015 provided remote physical rehabilitation through means of telerehabilitation for patients suffering from chronic pain disorders [11]. This study implemented partial exercise-based telerehabilitation in the intervention group, where the patients had to record an exercise in their own environment every week and receive a teleconference with the therapist the following week. The control group received on-site service as part of their rehabilitation program. The results of this study found significant results in both groups, however, low scores on satisfaction and usability in the intervention group were found. Only 13% of patients found the utility of an exercise-based telerehabilitation program to be “best imaginable” whereas 43% of patients found it to be “ok.” The results of the study indicate that a substantial method to increase motivation and utility is needed when implementing telerehabilitation methods in physiotherapy as only one-fifth of patients in the intervention group found the remote care to be motivating [11]. A systematic review analyzing the physical examination components adapted for telemedicine conducted by Lu et al. (2022) found that virtual assessments were similar to the in-person services in areas such as musculoskeletal exams and critical care [12]. However, the authors have found that the studies examining the effectiveness of telemedicine in health domains occupy a small sample size, making it difficult to draw large conclusions regarding the adaptation of telemedicine services.

The motivation and expansion of the use of telehealth and telerehabilitation methods lie within the hands of healthcare professionals. The attitudes healthcare professionals have regarding the effectiveness of telehealth and telerehabilitation services are critical to the accessibility patients have to these services [13]. In a systematic review assessing providers’ attitudes towards providing tele-mental health services through videoconferencing found overall positive attitudes across the studies [13]. The results of the review indicate providers found remote services to improve accessibility of care, be time-effective, and increase efficiency of services, despite drawbacks such as technical difficulties, increased workload in some instances, and barriers to therapeutic relationships [13].

Telerehabilitation can be an effective program to help workers with physical and or mental health disabilities [14]. The majority of studies exploring the effects of telerehabilitation have focused on patients’ and clinicians' perspectives on such programs. Additionally, telerehabilitation has become a necessity over the course of the pandemic by providing rehabilitation methods and programs remotely. Studies have found this method arguably as effective as face-to-face rehabilitation, especially with individuals returning to work [15]. However, there has not been strong directional evidence examining on the effectiveness of telerehabilitation in the literature. Thus, the purpose of this systematic review is to evaluate the effectiveness of telerehabilitation interventions on patients’ mental and physical outcomes.

## Methods

A systematic review and meta-analysis will be conducted to examine and analyze the impact of telerehabilitation on patient outcomes and following the Preferred Reporting Items of Systematic Reviews and Meta-Analyses (PRISMA) guidelines (PRISMA, 2015), (see Additional file 1). This systematic review has been registered with PROSPERO under registration number CRD42022297849. During the process of this review, there have been no reviews similar registered in PROSPERO. The Cochrane Handbook for Systematic Reviews also did not hold any reviews with a similar objective.

### Search strategy

The systematic review will use the following databases to examine the literature on telerehabilitation and patient outcomes: APA PsychINFO, Embase (Ovid), MEDLINE (Ovid), CINAHL, and Scopus. The search strategy was developed by the research team and in consultation with a University of Toronto health sciences librarian (Table 1). The search will include the use of text words and subject headings that relate to “telerehabilitation”, “occupational therapy”, “physical therapy” or “speech therapy”.

**Table 1:** Search strategies for each database

**Database**	Search strategy
Ovid MEDLINE	-(e-health or ehealth).tw,kf
	-exp Speech Therapy/
	-exp Occupational Therapy/
	-telemedicine/ or telerehabilitation/ or telehealth/ or telepsychiatry/
	-(Telemedicine or tele medicine or tele-medicine or telerehab* or telepsychiatr*).tw,kf
	-(Text messag* or video conferenc*).tw,kf
	-((online or web or remote* or virtual or digital) adj1 (intervention* or therap* or aftercare or rehab* or consult*)).tw,kf
	-occupational therapist/ or physical therapist/ or speech therapist/
	-exp physical therapy/
	-1 or 4 or 5 or 6 or 7
	-2 or 3 or 8 or 9
	-10 and 11
Embase	1. (e-health or ehealth).tw,kw
	2. exp Speech Therapy/
	3. exp Occupational Therapy/
	4. telemedicine/or telerehabilitation/or telehealth/or telepsychiatry/
	5. (Telemedicine or tele medicine or tele-medicine or telerehab* or telepsychiatr*).tw,kw
	6. (Text messag* or video conferenc*).tw,kw
	7. ((online or web or remote* or virtual or digital) adj1 (intervention* or therap* or aftercare or rehab* or consult*)).tw,kw
	8. occupational therapist/or physical therapist/or speech therapist/
	9. exp physical therapy/
	10. 1 or 4 or 5 or 6 or 7
	11. 2 or 3 or 8 or 9
	12. 10 and 11
APA PsycINFO	-(ehealth or e-health).ti,ab,id
	-exp Speech Therapy/
	-exp Occupational Therapy/
	-telemedicine/ or telerehabilitation/ or telehealth/ or telepsychiatry/
	-(Telemedicine or tele medicine or tele-medicine or telerehab* or telepsychiatr*).ti,ab,id
	-(Text messag* or video conferenc*).ti,ab,id
	-((online or web or remote* or virtual or digital) adj1 (intervention* or therap* or aftercare or rehab* or consult*)).ti,ab,id
	-occupational therapist/or physical therapist/or speech therapist/
	-exp physical therapy/
	-1 or 4 or 5 or 6 or 7
	-2 or 3 or 8 or 9
	-10 and 11
CINHAL	1. S1: (MH "Telemedicine + ") OR (MH "Telerehabilitation") OR (MH "Telepsychiatry") OR (MH "Telehealth") OR (MH "Text Messaging") OR (MH "Videoconferencing")
	2. S2: “e-health”
	3. S3: “ehealth”
	4. S4: (virtual N2 (therapy or care or rehab* or consult* or intervention)) OR (online N2(therapy or care or rehab* or consult* or intervention)) OR (remote N2 (therapy or care or rehab* or consult* or intervention)) OR (web N2 (therapy or care or rehab* or consult* or intervention)) OR (digital N2(therapy or care or rehab* or consult* or intervention))
	5. S5: (MH "Rehabilitation") OR (MH "Occupational Therapy") OR (MH "Occupational Therapy Service") OR (MH "Home Occupational Therapy") OR (MH "Occupational Therapy Practice") OR (MH "Occupational Therapy Assistants")
	6. S6: S1 OR S2 OR S3 OR S4
	7. S7: (MH "Physical Therapy") OR (MH "Physical Therapy Assessment") OR (MH "Physical Therapist Assistants") OR (MH "Home Physical Therapy") OR (MH "Physical Therapy Service")
	8. S8: physical therapist or occupational therapist or speech therapist
	9. S9: (MH "Speech and Language Assessment") OR (MH "Speech Therapy") OR (MH" Language Therapy") OR (MH "Alternative and Augmentative Communication") OR (MH" Rehabilitation, Speech and Language")
	10. S10: S5 OR S7 OR S8 OR S9
	11. S11: S6 and S10
SCOPUS	-“telemedicine” OR “telerehabilitation” OR “telehealth” OR “ehealth” OR “e-health” OR “telepsychiatry”
	-(Online or web or remote* or virtual or digital) w/1 (intervention* or therap* or aftercare or rehab* or consult*))
	-“Occupational therap*” OR “physical therap*” OR “speech therap*
	-#1 AND #2
	-#3 AND #4

#### Eligibility criteria

The inclusion criteria that this systematic review will use to determine the eligibility of studies are (i) peer-reviewed studies, (ii) a study population that includes workers 18 years of age or older who are absent from work due to a work-related physical injury or mental health condition, (iii) mental health conditions diagnosed by a psychiatrist and/or related health professional using the Diagnostic and Statistical Manual of Mental Disorders, Fifth Edition and the International Classification of Diseases 11th Revision, (iv) studies examining mental health conditions that include patients receiving drug therapy, (v) studies that implement and examine a telerehabilitation intervention, (vi) intervention(s) must be delivered by a licensed occupational therapist, physical therapist or speech language pathologist in their respective jurisdiction, (vii) studies that report the effectiveness of the intervention on mental or physical outcomes, (viii) study designs that will be considered for inclusion are randomized controlled trials, non-controlled trials with pre- and post- treatment measures, cohort studies, cross-sectional studies, mixed-method studies, longitudinal studies, observational studies, and retrospective studies, and (ix) studies must be reported in English.

The exclusion criteria is as follows: (i) non-peer-reviewed studies, (ii) knowledge syntheses (e.g., literature reviews, systematic reviews, scoping reviews), book chapters, and case studies will not be considered, (iii) study population is under the age of 18 years and older and who are absent from work for conditions that do not include mental or physical health, (iv) studies that do not examine telerehabilitation or similar interventions, and (v) studies that are not reported in English.

### Data collection/study selection

Two reviewers will analyze, collect, and report the data of articles that are retrieved. Studies that meet the inclusion criteria will be stored in Covidence, a commercially available web-based software for systematic review management [16]. Title and abstract examination will be done by the reviewers and those that meet the inclusion criteria stated above will move to the second screening phase. Full-text screening will be done independently by each reviewer to review the evidence from each study. All reviewers will be blinded during each screening stage. Any duplicates found during the title and abstract screening will be removed. Disagreements that arise from each phase of screening will be referred to the senior researchers for consideration. To calculate the interrater reliability among raters, the kappa statistic will be utilized for categorical variables [17]. The intraclass correlation coefficient will be used to test agreement between raters on continuous variables [18].

### Data extraction

Data extraction will be conducted by the reviewers with an outline provided to obtain data on the following (i) author name(s), (ii) year of publication, (iii) detailed description of the population of interest (i.e., physical and/or mental health condition, gender, age), (iv) study design, (v) description of telerehabilitation or similar interventions and how it will be implemented (i.e., who delivered the intervention), (vi) description of how mental and physical health outcomes are measured, and (vii) overall findings. This systematic review will provide a data analysis of the articles to be included. A table similar to the one developed by Kroon et al. (2014) will be presented summarizing the characteristics of the studies contributing to the synthesis. The table will illustrate the following frameworks: (i) study name; (ii) study design; (iii) characteristics of participants (age, diagnosis, job title); (iv) type of intervention; (v) control; and (vi) effect size to determine the strength of the interventions [19].

### Meta-analysis

The meta-analysis will be conducted using the Preferred Reporting Items for Systematic Review and Meta-Analysis (PRISMA) guidelines. The meta-analysis will investigate the impact and effectiveness of the telerehabilitation interventions on physical and mental health outcomes. Given the anticipated heterogeneity of data and the variability in intervention study design, a random-effects meta-analysis will be performed. Random effects models allow for statistical generalization beyond the studies included in the meta-analysis. Odds ratio (OR) and relative risk (RR) with 95% confidence intervals will be calculated for each study as a synthesized measure of effect size [20, 21]. Individual study and pooled effect sizes will be calculated and reported. An overall effect size will be calculated in studies with overlapping samples or subgroupings.

Based on data availability of at least 5 studies, meta-analyses will be performed for each physical and mental health outcomes identified. We anticipate performing separate meta-analyses for the following outcomes: pain reduction, orthopaedic outcomes, sleep quality, depression, and anxiety. Global meta-analyses for physical and mental-health outcomes will be reported if there are less than 5 studies examining the specified outcome. The overall effectiveness of the telerehabilitation intervention(s) will be assessed and compared to traditional rehabilitation interventions in each of the domains reported above. A moderator analysis will be performed to investigate the effects of moderating variables (e.g., sample composition, methodological characteristics) on the calculated effect size and heterogeneity of studies included.

Meta-analysis data will be reported graphically through forest and funnel plots to assess the dataset for any directional effects related to publication bias and potential outliers. Publication bias will also be assessed through Egger’s regression test. Sensitivity analyses will be conducted using Rosenthal’s Fail-Safe N and Duval and Tweedie’s trim and fill procedures. These analyses will be presented in a summary table to examine the robustness of the findings [22]. Heterogeneity will be measured using the *Q* and *I*^2^ statistic. A significant *Q* statistic indicates a rejection of the null hypothesis that no heterogeneity exists between and within the studies included. The *I*^2^ statistic indicates the degree of heterogeneity between the studies included where 0–20% represents low heterogeneity, 21–50% represents moderate heterogeneity, and > 51% represents substantial heterogeneity [23]. The outlined meta-analytical procedures will be performed using R statistical software.

### Risk of bias assessment

To assess the quality of the studies in the review, the Critical Appraisal Skills Programme (CASP) checklist (CASP UK, 2020) will appraise the quality and validity of each study. Two reviewers will be involved in the quality assessment using the CASP checklist to analyze included studies that are identified as randomized control trials, case controls, and cohort studies [24]. The Newcastle Ottawa scale will be used for cross-sectional studies [25]. Included studies will be classified by low, moderate, and high bias. The GRADE (Grading of Recommendations, Assessment, Development, and Evaluations) framework will be implemented to assess the quality of evidence from included studies (26). The GRADE approach comprises of five criteria: risk of bias, imprecision, inconsistency, indirectness, and publication bias [27]. The two reviewers will independently use this framework with the pooled studies. Disagreements will be resolved first between the two reviewers and if a consensus is not reached, then a third reviewer will be approached.

## Discussion

The systematic review will contribute to the literature surrounding telerehabilitation and patient outcomes from occupational therapy, physical therapy, and speech therapy. This knowledge synthesis will aim to present the findings from descriptive and statistical means to summarize and evaluate the effectiveness of telerehabilitation on patient mental and physical health outcomes. The utilization of telerehabilitation and similar telehealth treatments has increased throughout the COVID-19 pandemic. However, much is still unclear regarding the effectiveness of these methods in the delivery and service of healthcare, and their effect on health outcomes.

A systematic review and meta-analysis evaluating the management of musculoskeletal conditions using real-time telerehabilitation treatment compared to standard practice found telerehabilitation methods were as effective as face-to-face care, in areas such as physiotherapy [26]. The effectiveness of telerehabilitation programs with physiotherapy has been demonstrated by systematic reviews and a retrospective pre-post study. The results of these studies indicate quality of life and functioning (e.g., physical and cognitive) are improved through telerehabilitation interventions [27, 28]. Many studies examining the effects and feasibility of telerehabilitation methods have used self-report methods (e.g., questionnaires) to quantify outcomes. The use of self-report methods has not contributed to the determination of whether telerehabilitation approaches have been effective or not, rather it has increased the need for more methodological research needed to validate its effectiveness and reliability [22, 23, 27].

The cost-effectiveness of telerehabilitation methods has also been explored within the literature. A randomized controlled trial evaluating the clinical and cost-effectiveness of telerehabilitation for individuals with chronic low back pain found that telerehabilitation-based therapy was more affordable and effective than clinic-based therapy provided to participants [28]. Additionally, a cost-effectiveness analysis of cardiac telerehabilitation implemented with a traditional center-based cardiac rehabilitation was found to be significantly effective compared to the traditional in-person cardiac rehabilitation alone [22]. Nelson et al. (2021) concluded that telerehabilitation is more cost-effective and efficient than traditional care for total hip replacement patients in their trial-based economic evaluation [23].

The response to the pandemic has implemented many restrictions. To decrease the transmission of the COVID-19 virus, social distancing was implemented, and in-person activities were reduced or removed entirely. Patients and individuals that seek rehabilitative treatment have been faced with a challenge in receiving timely care and health care practitioners have had difficulties delivering timely, high-quality treatment. Current restrictions have also influenced economic activity and budgets within many healthcare sectors. These barriers have imposed difficulties on rehabilitation centers and other clinical practices, centers, and programs. The utilization of remote rehabilitation programs, treatments, and services provides quality, affordable, and accessible services for those that need them. Therefore, the aim of this study is to contribute to the literature surrounding telehealth methods and health outcomes by examining the effectiveness of telerehabilitation on patients mental and physical health outcomes.

The findings of this systematic review and meta-analysis will examine the state of the evidence of telerehabilitation on patient outcomes, with respect to telehealth treatments improving patients' mental and/or physical health. The results of the findings of this review will evoke interest among therapists, healthcare professionals and occupational researchers, and can be applied to areas of technology, mental health, physical health, occupational therapy, physical therapy, speech therapy, and similar practice areas. Acknowledging the feasibility of telerehabilitation interventions, researchers and healthcare professionals can leverage the current and future state of healthcare in patient populations. The assessment of the strength of the telerehabilitation interventions evaluated in this systematic review and meta-analysis will not only identify the effectiveness of different telehealth interventions for professionals to implement in their role but also provide the ability and interest for these professionals and researchers to conduct and further the research within this scope.

This systematic review and meta-analysis propose many strengths. This knowledge synthesis will conduct a risk of bias for each study, provide a detailed inclusion and exclusion criteria, and demonstrate a sufficient examination of each study. However, this review contains limitations. Studies that are not in English will be excluded from this review, which may restrict potential studies that are not able to contribute to the evidence base. Furthermore, there is the possibility of limited studies in which conclusions and quantitative data can be drawn, due to heterogeneity in study designs, demographic characteristics, measurement tools, and definitions of work performance and functioning [28]. Additionally, this review will only include studies with OTs, PTs, and SLPs, excluding data on other allied health professionals. However, excluding other allied health professionals will keep the scope of this review specific and comprehensive of the literature that exists. This systematic review and meta-analysis will strengthen the literature and knowledge around telerehabilitation and its impact on patient outcomes.

## Supplementary Information

**Additional file 1. **PRISMA-P (Preferred Reporting Items for Systematic review and Meta-Analysis Protocols) 2015 checklist: recommended items to address in a systematic review protocol* 

## Data Availability

Not applicable.
